# 1,4-Bis(4-chloro­phenyl­seleno)-2,5-di­methoxy­benzene

**DOI:** 10.1107/S1600536808039469

**Published:** 2008-12-03

**Authors:** Henning Osholm Sørensen, Nicolai Stuhr-Hansen

**Affiliations:** a Center for Fundamental Research: Metal Structures in Four Dimensions, Risø National Laboratory for Sustainable Energy, Technical University of Denmark, Frederiksborgvej 399, PO 49, DK-4000 Roskilde, Denmark; b Department of Medicinal Chemistry, University of Copenhagen, Universitetsparken 2, DK-2100 Copenhagen, Denmark

## Abstract

The title compound, C_20_H_16_Cl_2_O_2_Se_2_, utilizes the symmetry of the crystallographic inversion center. Mol­ecular chains are formed through symmetric C—H⋯Cl inter­actions around inversion centers, mimicking the commonly observed symmetric hydrogen-bonded dimer pattern often found in carboxylic acids.

## Related literature

For background to the electrophilic aryl­selenylation of reactive arenes, see: Santi *et al.* (2008 ▶); Nicolaou *et al.* (1979 ▶); Gassman *et al.* (1982 ▶); Yoshida *et al.* (1991 ▶); Tiecco *et al.* (1994 ▶); Engman & Eriksson (1996 ▶); Henriksen (1994 ▶); Henriksen & Stuhr-Hansen (1998 ▶). For related structures, see: Oddershede *et al.* (2003 ▶). For related supra­molecular patterns, see: Gavezzotti & Filippini (1994 ▶); Allen *et al.* (1999 ▶); Sørensen & Larsen (2003 ▶); Sørensen *et al.* (1999 ▶).
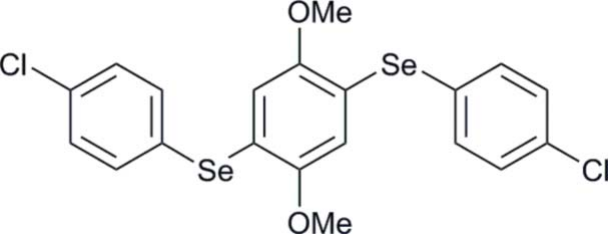


## Experimental

### 

#### Crystal data

C_20_H_16_Cl_2_O_2_Se_2_
*M* *_r_* = 517.15
Monoclinic, 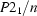

*a* = 11.7737 (17) Å
*b* = 6.6535 (6) Å
*c* = 13.438 (5) Å
β = 114.136 (16)°
*V* = 960.7 (4) Å^3^
*Z* = 2
Cu *K*α radiation
μ = 7.47 mm^−1^
*T* = 122 (1) K
0.44 × 0.15 × 0.13 mm

#### Data collection

Enraf–Nonius CAD-4 diffractometer
Absorption correction: gaussian (DeTitta, 1985 ▶). *T*_min_ = 0.242, *T*_max_ = 0.796
2641 measured reflections
1976 independent reflections
1919 reflections with *I* > 2σ(*I*)
*R*_int_ = 0.030
5 standard reflections frequency: 166.7 min intensity decay: 5.7%

#### Refinement

*R*[*F* ^2^ > 2σ(*F* ^2^)] = 0.028
*wR*(*F* ^2^) = 0.075
*S* = 1.10
1976 reflections
119 parameters
H-atom parameters constrained
Δρ_max_ = 0.59 e Å^−3^
Δρ_min_ = −1.20 e Å^−3^

### 

Data collection: *CAD-4 EXPRESS* (Enraf–Nonius, 1994 ▶); cell refinement: *CAD-4 EXPRESS*; data reduction: *DREAR* (Blessing, 1987 ▶); program(s) used to solve structure: *SHELXS97* (Sheldrick, 2008 ▶); program(s) used to refine structure: *SHELXL97* (Sheldrick, 2008 ▶); molecular graphics: *PLATON* (Spek, 2003 ▶); software used to prepare material for publication: *SHELXL97*.

## Supplementary Material

Crystal structure: contains datablocks global, I. DOI: 10.1107/S1600536808039469/sg2281sup1.cif

Structure factors: contains datablocks I. DOI: 10.1107/S1600536808039469/sg2281Isup2.hkl

Additional supplementary materials:  crystallographic information; 3D view; checkCIF report

## Figures and Tables

**Fig. 1.**
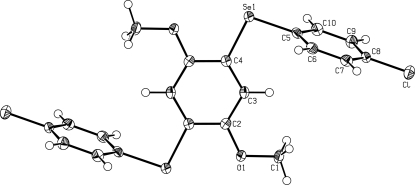
Thermal elipsoid plot of (1) including labelling of the atoms. The displacement ellipsoids are drawn at the 50% probability level. H atoms are shown as spheres with an arbitrary radii.

**Fig. 2.**
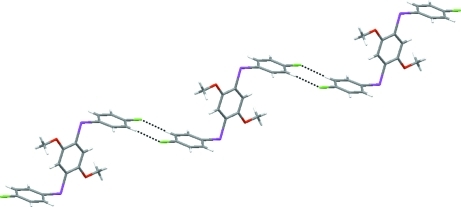
A view of the cyclic C—H···Cl interactions linking the molecules into a chain.

**Fig. 3.**
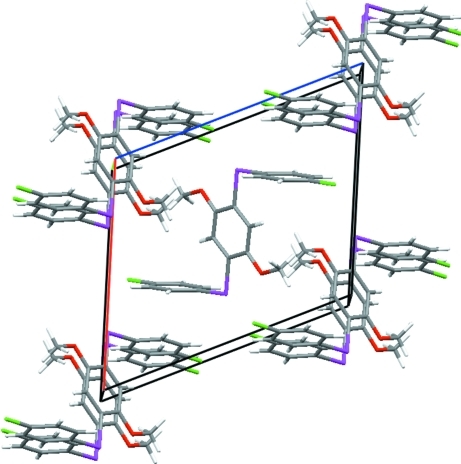
Packing diagram of viewed down the b axis.

## supplementary crystallographic information

### Comment

The structure of the title compound, shown in Fig. 1, crystallized in space group P 2_1_/n utilizing the crystallography inversion center in the molecular symmetry. Generally the molecular geometry of 1 is in close agreement with the related compound 1,3-dimethoxy-4,6-bis(phenylseleno)benzene, hereafter DMPSB. All bond distances and angles are the same within the experimental uncertainty. The molecular conformation of 1 is also very similar to the chloro-unsubstituted compound DMPSB having the planes of phenylseleno groups arranged perpendicular to the plane of the central benzene moiety, but rotated in opposite directions forming a *Z* like conformation (Fig. 1). Leading to the formation of intramolecular C_ar_—H···π interactions.

The molecular packing arrangement is dominated by molecular chains (see Fig. 2) formed by cyclic C_ar_—H···Cl interactions [H7···Cl_i_ = 2.96 Å, C7—H7···Cl_i_ = 166.0°; symmetry code: (i) 2 - *x*,1 - *y*,1 - *z*] around an inversion center leading to a pattern, which highly resembles the cyclic hydrogen-bonded dimers frequently observed in carboxylic acids. The C_ar_—H···Cl type of cyclic interaction found in 1 has also been observed in other compounds having a *p*-chlorosubstituted phenyl group, *e.g.* in the structure of racemic *p*-chlorophenoxypropionic acid, where the distance H···Cl is 2.92 Å [C—H···Cl 175°]. The chains are stacked such that the π-π interactions between the phenelseleno groups and between the benzene rings along the diagonal of the b and c-axes, respectively. Due to the chain formation in 1 the packing arrangement is rather different from the pattern found in DMPSB, where interactions with chlorine cannot be formed.

### Experimental

Crystals suitable for an X-ray diffraction experiment were obtained by slow crystallization from hot toluene.

### Refinement

Hydrogen atoms of (1) were found in the difference Fourier map. All hydrogen atoms were treated as riding atoms with C—H distances of 0.95 for C_ar_ and 0.98 for the C_Me_. Isotropic displacement parameters for all H atoms were constrained to 1.2*U*_eq_ of the connected non-hydrogen atom (1.5*U*_eq_ for Me groups).

### Crystal data

**Table:**

C_20_H_16_Cl_2_O_2_Se_2_	*F*(000) = 508
*M**_r_* = 517.15	*D*_x_ = 1.788 Mg m^−^^3^
Monoclinic, *P*2_1_/*n*	Melting point: 193 K
Hall symbol: -P 2yn	Cu *K*α radiation, λ = 1.54184 Å
*a* = 11.7737 (17) Å	Cell parameters from 20 reflections
*b* = 6.6535 (6) Å	θ = 39.3–40.7°
*c* = 13.438 (5) Å	µ = 7.47 mm^−^^1^
β = 114.136 (16)°	*T* = 122 K
*V* = 960.7 (4) Å^3^	Block, white
*Z* = 2	0.44 × 0.15 × 0.13 mm

### Data collection

**Table:**

Enraf–Nonius CAD-4 diffractometer	1919 reflections with *I* > 2σ(*I*)
Radiation source: fine-focus sealed tube	*R*_int_ = 0.030
graphite	θ_max_ = 74.9°, θ_min_ = 4.2°
ω–2θ scans	*h* = −14→14
Absorption correction: gaussian (DeTitta, 1985).	*k* = −7→8
*T*_min_ = 0.242, *T*_max_ = 0.796	*l* = 0→16
2641 measured reflections	5 standard reflections every 166.7 min
1976 independent reflections	intensity decay: 5.7%

### Refinement

**Table:**

Refinement on *F*^2^	Secondary atom site location: difference Fourier map
Least-squares matrix: full	Hydrogen site location: difference Fourier map
*R*[*F*^2^ > 2σ(*F*^2^)] = 0.028	H-atom parameters constrained
*wR*(*F*^2^) = 0.076	*w* = 1/[σ^2^(*F*_o_^2^) + (0.0433*P*)^2^ + 0.9774*P*] where *P* = (*F*_o_^2^ + 2*F*_c_^2^)/3
*S* = 1.10	(Δ/σ)_max_ < 0.001
1976 reflections	Δρ_max_ = 0.60 e Å^−^^3^
119 parameters	Δρ_min_ = −1.20 e Å^−^^3^
0 restraints	Extinction correction: *SHELXL97* (Sheldrick, 2008), Fc^*^=kFc[1+0.001xFc^2^λ^3^/sin(2θ)]^-1/4^
Primary atom site location: structure-invariant direct methods	Extinction coefficient: 0.0029 (3)

### Special details

**Table:**

Geometry. All e.s.d.'s (except the e.s.d. in the dihedral angle between two l.s. planes) are estimated using the full covariance matrix. The cell e.s.d.'s are taken into account individually in the estimation of e.s.d.'s in distances, angles and torsion angles; correlations between e.s.d.'s in cell parameters are only used when they are defined by crystal symmetry. An approximate (isotropic) treatment of cell e.s.d.'s is used for estimating e.s.d.'s involving l.s. planes.
Refinement. Refinement of *F*^2^ against ALL reflections. The weighted *R*-factor *wR* and goodness of fit *S* are based on *F*^2^, conventional *R*-factors *R* are based on *F*, with *F* set to zero for negative *F*^2^. The threshold expression of *F*^2^ > σ(*F*^2^) is used only for calculating *R*-factors(gt) *etc*. and is not relevant to the choice of reflections for refinement. *R*-factors based on *F*^2^ are statistically about twice as large as those based on *F*, and *R*- factors based on ALL data will be even larger.

### Fractional atomic coordinates and isotropic or equivalent isotropic displacement parameters (Å^2^)

**Table:**

	*x*	*y*	*z*	*U*_iso_*/*U*_eq_	
Se1	0.745362 (19)	0.19839 (3)	0.007848 (17)	0.01812 (12)	
Cl	1.00097 (5)	0.82041 (8)	0.40315 (5)	0.02427 (15)	
O1	1.23891 (13)	0.1213 (2)	0.13084 (12)	0.0192 (3)	
C1	1.2567 (2)	0.2869 (3)	0.20396 (19)	0.0232 (5)	
H1A	1.3459	0.3140	0.2431	0.035*	
H1B	1.2213	0.2535	0.2564	0.035*	
H1C	1.2150	0.4063	0.1623	0.035*	
C2	1.11846 (19)	0.0668 (3)	0.06767 (16)	0.0160 (4)	
C3	1.0144 (2)	0.1577 (3)	0.07253 (17)	0.0168 (4)	
H3	1.0245	0.2653	0.1219	0.020*	
C4	0.89557 (19)	0.0910 (3)	0.00514 (16)	0.0159 (4)	
C5	0.82080 (19)	0.3830 (3)	0.12694 (17)	0.0172 (4)	
C6	0.8614 (2)	0.3181 (3)	0.23448 (19)	0.0194 (4)	
H6	0.8506	0.1815	0.2493	0.023*	
C7	0.9173 (2)	0.4513 (3)	0.32023 (17)	0.0197 (4)	
H7	0.9457	0.4067	0.3936	0.024*	
C8	0.93109 (19)	0.6504 (3)	0.29699 (17)	0.0174 (4)	
C9	0.8891 (2)	0.7196 (3)	0.19057 (19)	0.0211 (5)	
H9	0.8985	0.8570	0.1762	0.025*	
C10	0.8331 (2)	0.5850 (3)	0.10537 (17)	0.0201 (4)	
H10	0.8030	0.6308	0.0321	0.024*	

### Atomic displacement parameters (Å^2^)

**Table:**

	*U*^11^	*U*^22^	*U*^33^	*U*^12^	*U*^13^	*U*^23^
Se1	0.01674 (16)	0.01956 (17)	0.01817 (16)	0.00021 (7)	0.00723 (11)	−0.00363 (7)
Cl	0.0267 (3)	0.0243 (3)	0.0206 (3)	−0.0041 (2)	0.0084 (2)	−0.00601 (18)
O1	0.0174 (7)	0.0196 (7)	0.0194 (7)	−0.0023 (6)	0.0063 (6)	−0.0059 (6)
C1	0.0242 (11)	0.0227 (11)	0.0217 (11)	−0.0045 (9)	0.0084 (9)	−0.0084 (8)
C2	0.0178 (9)	0.0154 (9)	0.0145 (9)	−0.0025 (8)	0.0064 (7)	0.0012 (7)
C3	0.0215 (10)	0.0136 (9)	0.0164 (9)	−0.0016 (8)	0.0089 (8)	−0.0014 (7)
C4	0.0192 (9)	0.0143 (9)	0.0156 (9)	0.0004 (7)	0.0084 (8)	0.0013 (7)
C5	0.0169 (9)	0.0178 (10)	0.0196 (10)	0.0013 (8)	0.0103 (8)	−0.0016 (8)
C6	0.0211 (10)	0.0160 (10)	0.0216 (11)	0.0022 (8)	0.0091 (9)	0.0024 (8)
C7	0.0208 (10)	0.0215 (11)	0.0170 (9)	0.0033 (8)	0.0080 (8)	0.0037 (8)
C8	0.0169 (9)	0.0173 (10)	0.0189 (10)	0.0001 (8)	0.0080 (8)	−0.0038 (8)
C9	0.0255 (11)	0.0163 (10)	0.0213 (11)	−0.0001 (8)	0.0093 (9)	0.0018 (8)
C10	0.0245 (11)	0.0196 (10)	0.0170 (10)	0.0017 (8)	0.0093 (8)	0.0031 (8)

### Geometric parameters (Å, °)

**Table:**

Se1—C4	1.921 (2)	C4—C2^i^	1.398 (3)
Se1—C5	1.923 (2)	C5—C6	1.393 (3)
Cl—C8	1.741 (2)	C5—C10	1.395 (3)
O1—C2	1.371 (2)	C6—C7	1.388 (3)
O1—C1	1.433 (2)	C6—H6	0.9500
C1—H1A	0.9800	C7—C8	1.386 (3)
C1—H1B	0.9800	C7—H7	0.9500
C1—H1C	0.9800	C8—C9	1.387 (3)
C2—C3	1.391 (3)	C9—C10	1.389 (3)
C2—C4^i^	1.398 (3)	C9—H9	0.9500
C3—C4	1.392 (3)	C10—H10	0.9500
C3—H3	0.9500		
			
C4—Se1—C5	97.92 (9)	C6—C5—Se1	120.74 (16)
C2—O1—C1	116.88 (17)	C10—C5—Se1	119.64 (16)
O1—C1—H1A	109.5	C7—C6—C5	120.6 (2)
O1—C1—H1B	109.5	C7—C6—H6	119.7
H1A—C1—H1B	109.5	C5—C6—H6	119.7
O1—C1—H1C	109.5	C8—C7—C6	118.9 (2)
H1A—C1—H1C	109.5	C8—C7—H7	120.6
H1B—C1—H1C	109.5	C6—C7—H7	120.6
O1—C2—C3	124.29 (19)	C7—C8—C9	121.7 (2)
O1—C2—C4^i^	115.37 (18)	C7—C8—Cl	119.75 (17)
C3—C2—C4^i^	120.34 (19)	C9—C8—Cl	118.60 (17)
C2—C3—C4	120.07 (19)	C8—C9—C10	119.0 (2)
C2—C3—H3	120.0	C8—C9—H9	120.5
C4—C3—H3	120.0	C10—C9—H9	120.5
C3—C4—C2^i^	119.60 (19)	C9—C10—C5	120.3 (2)
C3—C4—Se1	123.88 (16)	C9—C10—H10	119.9
C2^i^—C4—Se1	116.50 (15)	C5—C10—H10	119.9
C6—C5—C10	119.6 (2)		
			
C1—O1—C2—C3	−1.8 (3)	C10—C5—C6—C7	−2.0 (3)
C1—O1—C2—C4^i^	178.06 (18)	Se1—C5—C6—C7	179.10 (16)
O1—C2—C3—C4	−179.78 (19)	C5—C6—C7—C8	0.6 (3)
C4^i^—C2—C3—C4	0.4 (3)	C6—C7—C8—C9	0.8 (3)
C2—C3—C4—C2^i^	−0.4 (3)	C6—C7—C8—Cl	179.98 (16)
C2—C3—C4—Se1	177.83 (15)	C7—C8—C9—C10	−0.7 (3)
C5—Se1—C4—C3	−3.27 (19)	Cl—C8—C9—C10	−179.91 (17)
C5—Se1—C4—C2^i^	174.97 (16)	C8—C9—C10—C5	−0.8 (3)
C4—Se1—C5—C6	−83.67 (18)	C6—C5—C10—C9	2.1 (3)
C4—Se1—C5—C10	97.46 (18)	Se1—C5—C10—C9	−179.02 (17)

Symmetry codes: (i) −*x*+2, −*y*, −*z*.
